# Differential impact of parental region of birth on negative parenting behavior and its effects on child mental health: Results from a large sample of 6 to 11 year old school children in France

**DOI:** 10.1186/s12888-016-0832-7

**Published:** 2016-05-04

**Authors:** Viviane Kovess-Masfety, Mathilde Husky, Isabelle Pitrou, Christophe Fermanian, Taraneh Shojaei, Christine Chan Chee, Arjumand Siddiqi, Morton Beiser

**Affiliations:** Université Paris Descartes, Paris, EA 4057 France; EHESP, Avenue du Pr Leon Bernard, Rennes, 35043 France; Institut Pasteur, HAS, Paris, France; Screening and Prevention Bureau, SDS/DASES, Mairie de Paris, Paris, France; Department of Chronic Disease and Trauma, Institut de Veille Sanitaire, Saint-Maurice, France; University of Toronto Dalla Lana School of Public Health, Toronto, Canada; Department of Psychology, Ryerson University, Toronto, Canada

**Keywords:** Children, Internalizing disorders, Externalizing disorders, Mental health, Parenting styles, Ethno-cultural background

## Abstract

**Background:**

In France, one in 10 residents has immigrated mainly from North Africa, West Africa or the Caribbean including the French West Indies. However little is known about how parents from these regions behave when they migrate to countries that have different cultural norms. It is therefore important to determine how ethno-cultural background affects parental behavior and subsequent child mental health in the context of immigration. The objectives are: 1) to compare negative parenting behaviors of French residents from diverse ethno-cultural backgrounds 2) to examine the relationship between parental region of origin and child mental health, and 3) to investigate the extent to which ethno-cultural context moderates the effect of parenting styles on child mental health.

**Methods:**

A cross-sectional study was conducted in 2005 in 100 schools in South-East France. The Dominic Interactive and the parent-reported Strengths and Difficulties Questionnaire were used to assess child psychopathology. The Parent Behavior and Attitude Questionnaire was used to assess parenting styles. The final sample included data on 1,106 mother and child dyads.

**Results:**

Caring and punitive attitudes were significantly different across mothers as a function of region of origin. This association was stronger for punitive attitudes with the highest prevalence in the Caribbean/African group, while mothers from Maghreb were more similar to French natives. Differences in caring behaviors were similar though less pronounced. Among children of Maghrebian descent, punitive parenting was associated with an increased risk of internalizing disorders while this association was weaker among children of African and Afro-Caribbean descent.

**Conclusions:**

Parental region of origin is an important component of both parenting styles and their effect on child mental health. Interventions on parenting should consider both the region of origin and the differential impact of origin on the effect of parenting styles, thus allowing for a finer-grained focus on high-risk groups.

## Background

Parenting style can contribute to mental health problems among children, as well as to their emotional well-being in adolescence and into adulthood [1]. Parental ethno-cultural background and immigration status both have been shown to be important predictors of parenting style.

The great majority of the literature on the role of ethno-cultural background on parental attitudes and its effect on child mental health has originated from the U.S. and has compared African-American or Hispanic parents to Caucasian parents [2–5]. These studies have suggested that ethnicity acts as a mediator of the effect of parental behavior on child mental health. Specifically, punitive or authoritarian attitudes may have differential effects on anxiety or conduct problems depending on parental background. These differential effects may possibly be due to different norms and expectations that children develop, and to the co-existence of these negative behaviors with parental warmth [4]. These important findings, however, do not easily transfer to other countries including European countries where persons with a different ethno-cultural background are often the product of immigration of persons migrating from former overseas colonies. In France, one in 10 residents has immigrated mainly from Maghreb (North-African countries including Algeria, Morocco and Tunisia), West Africa (primarily including Burkina Faso, Ivory Coast, Mali, Niger, and Senegal) or the Caribbean including the French West Indies (Guadeloupe and Martinique) and French Guyana, and from the Reunion Island.

In fact, very few studies have been published on parental attitudes in these regions. However, certain patterns have been described. For instance, one study indicated that 30 % of African and 40 % of Caribbean parents reported that corporal punishment was necessary to raise and educate children, which was consistent with the proportion of self-reported experiences of physical abuse indicated by their children [6]. These findings were corroborated by a study of British Caribbean families who were described as harsh and demanding in their parenting style, with strict expectations that their children display obedience and respect, and frequent use of corporal punishment [7]. The normative nature of harsh punishment was further shown in Guyanese mothers [6, 8]. However, one comparative study of Arab countries suggested that Algerian parenting style was more permissive and authoritarian than what was observed in most other Arab countries, a specificity attributed to the influence of French colonization [9]. Taken together, these studies documented the predominance of authoritarian and harsh parenting styles in most of the Afro-Caribbean cultures, while some Maghrebian regions appear more permissive. However little is known about how parents from these regions behave when they migrate to countries that have different cultural norms. It is therefore important to determine how ethno-cultural background affects parental behavior and subsequent child mental health in the context of immigration.

Finally, studies examining the impact of ethno-cultural background on parenting styles have often focused on adolescents whereas one could argue that younger children might be an equally important target considering that the latter may be more amenable to interventions at a time when the negative effects of mental health problems on academic achievement may be more readily manageable. That being said, the assessment of mental health in primary school-age children is often performed through the use of parent and/or teacher reports, which are known to underestimate the prevalence of internalizing disorders. In order to resolve this issue, self-administered instruments such as the Dominic Interactive were developed. This computerized test allows for the assessment of both externalizing and internalizing disorders in children as young as 6 years old [10–13].

To our knowledge studies on the role of ethno-cultural background on child mental health have never explored children 6 to 11 years old, nor have they used both parent-reported and child-reported assessments of child mental health.

The objectives of the present study are to: 1) examine the association between parental ethno-cultural background and negative parenting behaviors (i.e.; excessive punitive behaviors, low involvement or caring, and low autonomy-promoting attitudes) in a sample of families from the Provence Alpes Cote d’Azur (PACA) region, 2) examine the association between parental ethno-cultural background and child mental health, and 3), investigate the extent to which ethno-cultural context moderates the effect of parenting styles on child mental health.

## Methods

### Sample

The study sample is the result of a two-stage probability sampling strategy. First, 100 primary schools in the PACA region were randomly sampled. The PACA region encompasses a number of departments from Lyon to the Mediterranean Sea, and from the Rhone River to the French Alps. Its population is approximately 5 million residents including 10 % of immigrants. Second, 25 children were randomly selected within each school (5 from each grade within first to fifth grade) to ensure representativeness across the 1,856 schools of the area (serving approximately 296,257 pupils). Schools were stratified according to the following characteristics: public/private, rural/urban and Disadvantaged School Area DSA/not DSA. DSAs are defined by the Ministry of Education on the basis of low socio-economic status, low educational level and neighborhoods with high unemployment rates. Among the 100 primary schools selected, 99 agreed to participate. Some schools, mostly in isolated rural zones, had fewer than 25 children enrolled. Consequently, contacts were achieved for 2,341 children instead of 2500, among whom 2,324 met inclusion criteria for the 6 to 11 age range.

At least two weeks prior to the study, a letter of information containing a refusal form to return was mailed to the parents. On the day of the study, children whose parents’ had declined to participate were excluded and were not replaced. Children whose parents’ consented completed a computerized questionnaire on site; a packet was given to the child containing a questionnaire to be completed by one of his parents to establish socio-demographic characteristics, parental attitudes, and child psychopathology with an envelope to be sealed and directly returned to the school to a dedicated mail box.

Among the 2,341 eligible children, 19.7 % (*n* =462) of parents declined participation, and an additional 22.7 % (*n* =531) did not return their questionnaires. Overall, 3.8 % (*n* = 88) of the children were absent from school on the day of the study, and the data stemming from 24 child computerized questionnaires were compromised. As a result, the response rate for parent questionnaires was 72.5 % among those who participated; 54.4 % for the three informants questionnaires. Of the 1,291 parent questionnaires, 1,122 (87.0 %) were completed by mothers; the present study focused on mother-respondents only since gender could influence certain results and the father respondent group was too small to allow cultural group comparisons. As a result, the final total sample consisted of 1,106 mothers and child dyads.

### Socio-demographic variables

Parent questionnaires included questions on the following socio-demographic characteristics concerning each parent: parental region of birth, parental education level (highest level among parents), employment status (unemployment of either parent), household monthly income (amount per person), and family structure (single- or two-parent home).

### Ethno-cultural background

Each parent’s self-reported region of birth was used to create three distinct groups covering persons born in: the Caribbean or Africa (French West-Indies, or Sub-Saharan Africa), Maghreb, or Western regions. Sixteen parental couples in which one or both parents were born in Asia were excluded from the study as this sample was too small to permit meaningful analyses. According to the U.N., an immigrant is someone who was “born outside (his/her current) country of residence”. France’s former colonies are the major source of immigration and primarily include Maghreb (29 %), Sub-Saharan Africa (13 %), the French West Indies (Guadeloupe and Martinique) and the Reunion Island. Although these individuals would be considered as immigrants by the U.N., and come from vastly different ethno-cultural backgrounds, many are French citizens by virtue of their country’s colonial past or, as is the case for persons from the French West Indies, were living in what is still considered a region of France. However, the present study made the distinction between those born outside of metropolitan France (including former colonies or current oversees French territories), regardless of citizenship vs. those born in metropolitan France or in other Western countries.

The children were divided into 6 mutually exclusive groups combining each parent’s region of birth. The categories were defined as follows: both parents from either of the three regions, or one parent from either the Caribbean/Africa or Maghreb and the other from any other region, and one parent from a Western region while the other parent’s region of birth is undisclosed (Table 1).

**Table 1:** Socio-demographic characteristics of the sample by parental region of birth

	Parental region of birth							
*%* (*n*)	Both from Western region	Both from C/African region	Both from Mahrebian region	One from C/African region/One from other region	One from Maghrebian region/One from other region	One from Western region/Missing information on other parent	Total	*p*-value ^a^
*Sample size range by socio-demographic characteristic*	*660 - 778*	*16 - 25*	*33 - 39*	*37 - 54*	*67 - 81*	*112 - 129*	*925 - 1106*	
**Child’s gender**								
Boy	50.6 (394)	48.0 (12)	46.2 (18)	55.6 (30)	45.7 (37)	45.0 (58)	49.6 (549)	0.712
Girl	49.4 (384)	52.0 (13)	53.8 (21)	44.4 (24)	53.4 (44)	55.0 (71)	50.4 (557)	
**Child’s age, years**								
Mean (SD)	8.03 (1.47)	8.24 (1.27)	8.36 (1.65)	8.07 (1.65)	8.22 (1.52)	8.41 (1.49)	8.11 (1.49)	0.105
6–8 years	61.6 (478)	68.0 (17)	48.7 (19)	63.0 (34)	55.6 (45)	50.0 (64)	59.6 (657)	0.088
9–11 years	38.4 (298)	32.0 (8)	51.3 (20)	37.0 (20)	44.4 (36)	50.0 (64)	40.4 (446)	
**Number of children in household**								
2 or 3	78.7 (612)	52.0 (13)	46.2 (18)	64.8 (35)	66.7 (54)	55.8 (72)	72.7 (804)	**<1 E-10**
single child	15.2 (118)	8.0 (2)	2.6 (1)	13.0 (7)	18.5 (15)	38.0 (49)	17.4 (192)	
>3	6.2 (48)	40.0 (10)	51.3 (20)	22.2 (12)	14.8 (12)	6.2 (8)	10.0 (110)	
**Parental sociodemographic factors**								
Single-parent home	2.4 (19)	0 (0)	2.6 (1)	25.9 (14)	16.1 (13)	86.1 (111)	14.3 (158)	**<0.001**
**Monthly income (per person)**								
High	40.2 (283)	4.4 (1)	5.9 (2)	23.4 (11)	32.0 (24)	3.3 (4)	32.3 (325)	**<1 E-10**
Medium	35.7 (251)	17.4 (4)	20.6 (7)	25.5 (12)	24.0 (18)	33.6 (41)	33.1 (333)	
Low	24.2 (170)	78.3 (18)	73.5 (25)	51.1 (24)	44.0 (33)	63.1 (77)	34.5 (347)	
**Educationl level**								
High	43.4 (337)	16.0 (4)	5.3 (2)	30.2 (16)	35.0 (28)	11.6 (15)	36.5 (402)	**<1 E-10**
Medium	29.1 (226)	16.0 (4)	23.7 (9)	20.8 (11)	25.0 (20)	26.4 (34)	27.6 (304)	
Low	27.5 (213)	68.0 (17)	71.1 (27)	49.1 (26)	40.0 (32)	62.0 (80)	35.9 (395)	
**Employment status**								
Unemployment of at least one parent	9.9 (77)	36.0 (9)	37.8 (14)	18.0 (9)	17.7 (14)	12.9 (16)	12.7 (139)	**<0.001**
**Parental psychopathology**								
Mother’s psychological distress (MH5)	16.1 (106)	31.3 (5)	33.3 (11)	24.3 (9)	29.9 (20)	26.8 (30)	19.6 (181)	**0.001**
**Environmental factors**								
Living in a DSA	5.7 (44)	36.0 (9)	38.5 (15)	25.9 (14)	11.1 (9)	12.4 (16)	9.7 (107)	**<0.001**
Living in a rural setting	18.3 (142)	8.0 (2)	2.6 (1)	7.4 (4)	2.5 (2)	16.3 (21)	15.6 (172)	**<0.001**

^a^Unless otherwise specified, significance of Fisher’s exact test; for the number of children in household, income level and education level, the test is the

Freeman-Halton extension of Fisher’s test with a Monte-Carlo random sampling (10,000 samples). For age as a continuous variable, the p-value is from an ANOVA. Due to missing data for some socio-demographic characteristics, the sample size range is presented. Bold means significant 0.05 and above

### Parenting style and behavior

The parent questionnaire included the Parent Behavior and Attitude Questionnaire (PBAQ), an instrument adapted from the Vineland adaptive behavior scales, and previously used in several Canadian population-based surveys [14, 15]. The PBAQ assesses the frequency of specific behaviors in the previous six months and includes: involvement and caring, punitive behaviors, and autonomy-promoting attitudes.

Based on the description of optimal parenting provided by Belsky [16], negative parenting behaviors were defined as follows: excessive punishment, low caring and involvement behaviors, and low autonomy-promoting behaviors.

### Parental psychological distress

Parental psychological distress was assessed using the MH-5 scale of the Short-Form-36 (www.SF-36.org).

### Parent-reported child psychopathology

Child psychopathology was examined using the validated French version of the parent-reported Strengths and Difficulties Questionnaire (SDQ) [17]. The SDQ is a 25-item screening questionnaire for children from 4 to 16 years old. It comprises five subscales (containing five items each): emotional problems, conduct problems, hyperactivity-inattention, peer relationship problems, and prosocial behaviors. “Total difficulties” is the sum of the four problem behavior scales listed above [18].

### Child self-reported psychopathology

Each child completed the Dominic Interactive (DI) [10–13]. The DI is a self-administered computerized questionnaire for children ages 6 to 11. Children are invited to follow a cartoon-like character named Dominic, which can be adapted to match the child’s gender and ethnicity. The child is asked whether he or she feels the same as Dominic in 91 specific situations at home, at school and with or without other children. The DI situations were designed to depict the emotional and behavioral symptoms of seven common DSM-IV childhood mental disorders: Attention Deficit/Hyperactivity Disorder (ADHD), Conduct Disorder, Oppositional Defiant Disorder, Phobias, Separation Anxiety Disorder, Generalized Anxiety Disorder, and Major Depressive Disorder). The DI scores represent the child’s probability of suffering from an internalizing or externalizing disorder. This instrument has been shown to have a high positive predictive value in the identification of internalizing disorders [19].

### Statistical analysis

Three-class monthly income was derived from tertiles of the distribution of the per capita monthly income of the family (sum of net income of all persons in the household). Parental educational level was defined as: low for an educational level below high school graduation; medium for high school graduates; or high for those with a college education or above.

For most class variables, differences among region of birth categories were tested using Fisher’s exact test. For the number of children in the household, income, and education level, we used the Freeman-Halton extension of Fisher’s test with a Monte-Carlo random sampling (10,000 samples).

Statistical analyses were performed using Stata 11.2 (Stata Corporation, College Station, TX). Univariate and multivariate logistic regressions were used to examine the associations between negative parental behaviors, socio-demographic characteristics and child mental health (both SDQ and DI). The potential confounders and mediators (child age and gender, socioeconomic status, educational level, parental psychological distress) and the variables with p-values of 0.20 or below in the univariate analyses were included a priori in the multivariate logistic regression models (adjusted models). Missing data were multiply imputed using Stata’s *ice* package, generating 100 imputed datasets per original dataset. Interaction terms were created manually from the imputed ad hoc variables, when needed. Imputed data were analyzed using Stata’s *mi estimate* commands, and tests of estimates were performed on unrestricted fraction missing information (FMI) models.

## Results

Table 1 presents the socio-demographic characteristics of the sample by parental region of birth, taking into account whether both parents or one parent was from each region of interest. Major differences were observed. Few parents from Western regions had more than three children. Compared to families of other origins, families from western regions had a higher level of education, higher income, were more likely to be employed, lived in more affluent neighborhoods, and in urban rather than rural settings. Mothers born in non-western regions had higher levels of psychological distress than their Western counterparts. The rate of single-parent homes was higher among families with mixed origins as well as in families in which partner’s region of birth was missing.

Table 2 reports the prevalence of the three parental behaviors by maternal and paternal region of birth. Low caring and punitive behaviors varied significantly across maternal region of birth though this was more accentuated for punitive behaviors, the prevalence being much higher in the Caribbean/African group, while mothers from Maghreb were closer to Western mothers. The differences in caring behavior were similar but less pronounced. For autonomy promoting behaviors, no significant differences were found across region of birth, though a similar trend was noticeable.

**Table 2:** Prevalence of parenting behaviors by parental region of birth

*%* (*n*)		Father’s region of birth					
Low caring behavior		Western region	C/African region	Maghrebian region	Missing	Total	*p-value*
**Mother**	Western region	12.2 (777)	13.6 (22)	13.3 (45)	19.2 (125)	13.2 (969)	0.198
	C/African region	0 (15)	29.2 (24)	0 (2)	41.7 (12)	22.6 (53)	**0.023**
	Maghrebian region	15.4 (26)	- (0)	13.9 (36)	22.2 (9)	15.5 (71)	0.810
	Missing	0 (1)	- (0)	100 (1)	100 (1)	66.7 (3)	1.000
	**Total**	12.1 (819)	21.7 (46)	14.3 (84)	21.8 (147)	14.0 (1096)	**0.008**
	*p-value*	0.388	0.289	0.239	0.079	**0.022**	
**Low autonomy-promoting behavior**							
**Mother**	Western region	14.7 (770)	13.6 (22)	13.6 (44)	12.6 (127)	14.3 (963)	0.950
	C/African region	20.0 (15)	32.0 (25)	0 (2)	23.1 (13)	25.5 (55)	0.846
	Maghrebian region	19.2 (26)	- (0)	15.4 (39)	11.1 (9)	16.2 (74)	0.906
	Missing	100.0 (1)	- (0)	0 (1)	0 (1)	33.3 (3)	1.000
	**Total**	15.0 (812)	23.4 (47)	14.0 (86)	13.3 (150)	15.1 (1095)	0.407
	*p-value*	0.154	0.179	1.000	0.523	0.085	
**Punitive behavior**							
**Mother**	Western region	13.3 (755)	4.6 (22)	16.3 (43)	15.1 (126)	13.4 (946)	0.570
	C/African region	26.7 (15)	32.0 (25)	50.0 (2)	40.0 (10)	32.7 (52)	0.840
	Maghrebian region	7.7 (26)	- (0)	18.9 (37)	37.5 (8)	16.9 (71)	0.111
	Missing	0 (1)	- (0)	0 (1)	0 (1)	0 (3)	-
	**Total**	13.3 (797)	19.2 (47)	18.1 (83)	17.9 (145)	14.6 (1072)	0.226
	*p-value*	0.304	**0.025**	0.481	0.062	**0.004**	

Note: p-values are from linkage (exact Fischer) tests between negative attitudes and resp. mother’s birth place (in columns) and father’s birth place (in rows). Results reported as % (*n*). Bold means significant 0.05 and above

Among the mothers whose partner’s origin was missing, mothers from the Caribbean/Africa reported low caring behavior and punitive attitudes more frequently though these differences did not reach significance (*p* = .079 and *p* = .062, respectively).

The differences were mainly present for the group with both parents from Caribbean or African regions where the prevalence of low caring was very high. No differences were observed with regard to the partner’s region of origin with the exception of low caring behaviors, again more prevalent in the Caribbean/African partner group and for the group for which information on the origin of the father was missing.

Table 3 reports child mental health status by parental region of birth. Conduct disorders as reported by the mother were more frequent in the Caribbean/African group, followed by the group from Maghreb as compared to the group from Western regions. Peer relationship difficulties were elevated in children of Maghrebian descent while this did not hold true for the Caribbean/African group whose report of these difficulties was even lower than in the Western group. The group from Maghreb had the highest proportion of children with ‘total difficulties’ on the SDQ. Between-group comparisons of the frequency of SDQ internalizing disorders did not reach significance (*p* = .064) although children of parents from the Caribbean/Africa and Maghreb had a higher percentage when compared to children of parents from the West. Similar results were obtained with child-reported internalizing disorders (*p* = .057). Discrepancies were observed between parent- and child- reported conduct disorders. The comparisons were significant for parent-report conduct disorders (*p* = .001) but they were not for child-reported conduct disorders.

**Table 3:** Child mental health status by parental region of birth

	Parental region of birth							
*%* (*n*)	Both from Western region	Both from C/African region	Both from Mahrebian region	One from C/African region/One from other region	One from Maghrebian region/One from other region	One from Western region/Missing information on other parent	Total	*p*-value^a^
Parent-reported child psychopathology (SDQ)								
* Sample size range)*	*775*	*25*	*39*	*51*	*80*	*128–129*	*1098–1099*	
* Internalizing disorders*	9.2 (71)	20.0 (5)	18.0 (7)	13.7 (7)	16.3 (13)	11.6 (15)	10.7 (118)	0.064
CD	9.8 (76)	32.0 (8)	20.5 (8)	7.8 (4)	16.3 (13)	18.6 (24)	12.1 (133)	**0.001**
ADHD	11.9 (92)	12.0 (3)	5.1 (2)	7.8 (4)	8.8 (7)	14.1 (18)	11.5 (126)	0.644
Peer relationship difficulties	13.3 (103)	8.0 (2)	23.1 (9)	13.7 (7)	21.3 (17)	24.0 (31)	15.4 (169)	**0.012**
Total difficulties	7.0 (54)	12.0 (3)	18.0 (7)	7.8 (4)	7.5 (6)	16.4 (21)	8.7 (95)	**0.005**
* Externalizing disorders*	17.2 (133)	32.0 (8)	20.5 (8)	13.7 (7)	20.0 (16)	24.0 (31)	18.5 (203)	0.179
Child self-reported psychopathology (Dominic interactive)								
* Sample size*	*778*	*25*	*39*	*54*	*81*	*129*	*1106*	
GAD	9.9 (77)	20.0 (5)	7.7 (3)	3.7 (2)	12.4 (10)	14.0 (18)	10.4 (115)	0.159
SAD	12.0 (93)	20.0 (5)	15.4 (6)	11.1 (6)	17.3 (14)	17.1 (22)	13.2 (146)	0.321
SPh	11.1 (86)	20.0 (5)	18.0 (7)	7.4 (4)	8.6 (7)	16.3 (21)	11.8 (130)	0.160
MDD	8.5 (66)	16.0 (4)	10.3 (4)	3.7 (2)	9.9 (8)	10.9 (14)	8.9 (98)	0.432
CD	9.4 (73)	8.0 (2)	12.8 (5)	3.7 (2)	7.4 (6)	10.9 (14)	9.2 (102)	0.637
ODD	10.5 (82)	8.0 (2)	15.4 (6)	3.7 (2)	9.9 (8)	7.8 (10)	10.0 (110)	0.450
ADHD	8.4 (65)	20.0 (5)	10.3 (4)	3.7 (2)	12.4 (10)	9.3 (12)	8.9 (98)	0.193
* Internalizing disorders*	19.4 (151)	32.0 (8)	30.8 (12)	16.7 (9)	25.9 (21)	27.9 (36)	21.4 (237)	0.057
* Externalizing disorders*	14.4 (112)	20.0 (5)	23.1 (9)	7.4 (4)	21.0 (17)	14.0 (18)	14.9 (165)	0.161

^a^significance of Fisher’s exact test across birthplace categories

Abbreviations: *CD* conduct disorder, *ADHD* attention deficit-hyperactivity disorder, *GAD* generalized anxiety disorder, *SAD* separation anxiety disorder, *SPh* specific phobia, *MDD* major depressive disorder, *ODD* oppositional defiant disorder

Table 4 presents the determinants of low caring, low autonomy promoting, and punitive behaviors in univariate and multivariate models controlling for the effects of the main socio-demographic determinants, the mother’s region of birth, and homogeneity of the couple’s origin. The child’s gender was a determinant of parental behavior. Low autonomy-promoting behaviors were more frequent for boys. The child’s age was also an important determinant. Being older was associated with higher probability of low caring attitudes, fewer low autonomy-promoting and punitive attitudes. Being from a large family was associated with lower caring. Maternal psychological distress was associated with lower caring and higher punitive attitudes, but not with fewer autonomy-promoting behaviors. Living in a rural area was associated with lower caring.

**Table 4:** Predictors of negative parental behaviors

	Low caring				Low autonomy-promoting				Punitive			
	Crude		Adjusted		Crude		Adjusted		Crude		Adjusted	
	OR	*P > z*	OR	*P > t*	OR	*P > z*	OR	*P > t*	OR	*P > z*	OR	*P > t*
Boy/girl	1.19	*0.317*	1.34	0.108	**2.02**	***<0.001***	**2.01**	***<0.001***	1.26	*0.185*	1.37	0.081
Child age > = 9 years/less than 9 years	**1.50**	***0.022***	**1.49**	**0.029**	**0.35**	***<0.001***	**0.33**	***<0.001***	**0.69**	***0.043***	**0.66**	**0.029**
***Number of children in household***												
single/2 or 3	0.65	*0.115*	0.58	0.059	0.87	*0.570*	0.93	0.781	0.77	*0.304*	0.76	0.285
more than 3/2 or 3	**2.52**	***<0.001***	**2.08**	**0.006**	1.44	*0.157*	1.49	0.171	1.29	*0.350*	0.94	0.846
Living apart from father yes/no	1.49	*0.085*	0.67	0.356	0.88	*0.605*	1.06	0.902	1.30	*0.271*	0.99	0.986
***Income***												
medium/low	**0.61**	***0.020***	0.70	0.128	0.91	*0.648*	1.06	0.806	0.75	*0.182*	1.02	0.941
high/low	**0.40**	***<0.001***	**0.56**	**0.053**	0.96	*0.832*	1.14	0.643	**0.64**	***0.046***	0.99	0.985
***Education level***												
medium/low	**0.55**	***0.007***	0.77	0.293	0.69	*0.089*	0.69	0.120	0.72	*0.129*	0.89	0.631
high/low	**0.48**	***<0.001***	0.76	0.283	0.80	*0.244*	0.71	0.152	**0.59**	***0.011***	0.72	0.191
At least one parent unemployed yes/no	1.36	*0.212*	0.91	0.740	0.83	*0.488*	0.74	0.302	**1.81**	***0.010***	1.33	0.264
Mother psychological distress yes/no	**1.86**	***0.004***	**1.67**	**0.029**	0.76	0.266	0.84	0.521	**2.48**	***<0.001***	**2.35**	***<0.001***
Mother’s region of birth												
Caribean Africa/Western	1.92	*0.056*	1.13	0.748	**2.04**	***0.027***	1.99	0.064	**3.13**	***<0.001***	**2.40**	**0.015**
Maghreb/Western	1.20	*0.586*	0.83	0.626	1.16	*0.657*	1.21	0.615	1.31	*0.412*	1.04	0.919
Homogeneity of couple												
Yes/no	1.09	*0.775*	1.22	0.560	0.97	*0.920*	1.08	0.802	1.02	*0.958*	1.34	0.361
Unknown/no	**2.01**	***0.051***	2.51	0.074	0.84	*0.623*	0.94	0.908	1.37	*0.376*	1.54	0.403
Living in a DSA yes/no	1.51	*0.129*	0.91	0.765	1.08	*0.791*	0.82	0.531	**1.74**	***0.036***	1.20	0.543
Rural environment yes/no	**1.64**	***0.022***	**1.62**	**0.037**	0.97	*0.901*	1.03	0.896	0.75	*0.270*	0.76	0.294

Bold means significant 0.05 and above

After controlling for these factors in multivariate analyses, region of birth remained a strong predictor of parental behavior. Mothers from the Caribbean/Africa were more punitive and slightly less autonomy promoting (though the latter did not reach significance). Homogeneity of the couple’s origins did not seem to influence parental behavior. However, in the case of missing information for the partner’s region of birth, the risk for lower caring was higher.

Table 5 presents multivariate models predicting child mental health status based on parent-report and child self-report. Gender was an important predictor of externalizing disorders and total difficulties with boys at higher risk than girls, while there were no gender differences for internalizing disorders. Maternal psychological distress was strongly associated with any child mental health problems though it was not associated with child mental health as reported by the child.

**Table 5:** Interaction between parental behavior, region of birth and child mental health

	Child mental health status									
	Parent-reported						Child self-reported			
	SDQ total difficulties		SDQ Conduct Disorder		internalizing disorders		Internalizing disorder			
							Model 1		Model 2	
	AOR	P > t	AOR	P > t	AOR	P > t	AOR	P > t	AOR	P > t
Boy/girl	**3.08**	**<0.001**	**1.98**	**0.001**	1.03	0.880	0.82	0.197	0.81	0.179
Child age > =9 years/less than 9 years	1.01	0.971	1.01	0.954	0.97	0.874	0.73	0.053	0.75	0.073
***Number of children in household***										
single/2 or 3	1.57	0.130	1.08	0.782	0.61	0.125	1.39	0.097	1.43	0.075
more than 3/2 or 3	0.75	0.520	0.74	0.409	1.21	0.558	0.65	0.143	0.73	0.264
***Income***										
medium/low	1.00	0.995	0.72	0.236	1.35	0.278	0.95	0.821	0.97	0.892
high/low	1.12	0.773	0.72	0.332	1.10	0.790	0.95	0.832	0.96	0.882
***Education level***										
medium/low	1.12	0.723	0.91	0.741	0.86	0.588	0.78	0.202	0.78	0.212
high/low	0.64	0.199	1.13	0.667	0.72	0.250	**0.48**	**0.001**	**0.47**	**0.001**
At least one parent unemployed yes/no	1.22	0.554	1.01	0.979	1.31	0.359	1.08	0.741	1.09	0.710
Mother psychological distress yes/no	**4.61**	**<0.001**	**2.93**	**<0.001**	**3.99**	**<0.001**	1.16	0.483	1.13	0.569
Mother’s region of birth										
CA/Africa/Western	1.01	0.985	1.19	0.685	1.19	0.748	0.84	0.658	1.41	0.393
Maghreb/Western	1.55	0.337	1.74	0.153	0.62	0.327	0.58	0.146	0.92	0.813
Homogeneity of couple										
No/yes	0.45	0.127	0.64	0.256	1.35	0.367	1.12	0.657	1.10	0.724
Missing/yes	**2.01**	**0.024**	1.66	0.077	1.10	0.769	0.96	0.854	0.96	0.877
Caring	1.42	0.251	**2.36**	**0.001**	0.98	0.954	0.96	0.871	0.94	0.801
Autonomy-promoting	0.81	0.561	**1.83**	**0.024**	0.80	0.496	0.95	0.820	1.06	0.793
Punitive behavior	**3.73**	**<0.001**	**4.96**	**<0.001**	0.94	0.855	1.43	0.084	**1.74**	**0.012**
“Region of birth x autonomy-promoting” interactions										
Caribean/Africa							1.20	0.813		
Maghreb							**4.16**	**0.054**		
“Region of birth x punitive” interactions										
Caribean/Africa					1.74	0.501			**0.19**	**0.034**
Maghreb					**5.06**	**0.056**			0.33	0.206
Living in a DSA yes/no	1.38	0.400	0.71	0.357	1.39	0.326	**1.95**	**0.007**	**2.04**	**0.004**
Rural environment yes/no	1.46	0.231	0.77	0.389	1.15	0.614	0.68	0.093	0.68	0.085

“living apart from father” (single-parent home) covariate was removed because it was correlated to “homogeneity of the couple” graded as yes, no or unknown. Bold means significant 0.05 and above

Missing information regarding the partner’s origin was associated with parent-reported total difficulties. Living in a disadvantaged environment was associated with internalizing disorders and higher education level of parents was a protective factor for that association. The three negative parental behaviors were strongly associated with conduct disorders as evaluated by the mother. However, only punitive behaviors were linked with internalizing disorders as reported by the child.

Maternal region of birth was not associated with any of the mental health problems once psychological distress was controlled for, along with socio-demographic variables. However, some interactions between parental behaviors and place of birth were observed. There was a slight interaction between internalizing disorders and punitive behaviors for mothers from Maghreb.

The percentage of children suffering from parent-reported internalizing disorders and whose mother was highly punitive was very high, whereas the percentage was very low when punitive behaviors were low. Low autonomy promoting attitudes in couples in which one is from Maghreb are associated with significant increases in child-reported internalizing disorders. Regarding punitive behaviors, originating from Caribbean/African regions was a protective factor for internalizing disorders as measured by the child. In other words, children whose mother has high punitive behaviors did not have more internalizing disorders if the mother originated from these regions, as opposed to what is observed in children of mothers from other origins. An interaction was also present for low autonomy-promoting attitudes in mothers from Maghreb for internalizing disorder as reported by the child. Frequent use of punitive attitudes was also significantly associated with increases in parent-reported internalizing disorders in couples from Maghreb.

## Discussion

The findings of the present study indicate that child mental health and parental behaviors vary as a function of parental region of origin. Based on self-reported data, mothers of Afro-Caribbean or African origin are more punitive and this holds true when the potential confounding effects of income, education, and psychological distress are controlled for, variables which prior studies have suggested are associated with parental warmth and disciplinary behaviors [6]. This finding is in line with existing research suggesting that Caribbean parents often rely on control and harsh disciplinary measures and display less nurturance towards their children as compared to what is observed in parents of European American descent [20]. This has also been shown in African-American parents as compared to European American parents [21]. In contrast, parents from Algeria, a region representing the bulk of Maghrebian immigrants living in France have been shown to display a mixed pattern of behavior with relatively high authoritative behaviors and an overall permissive parenting style, a pattern suggested to be linked to the prolonged exposure of Algerians to French culture [9].

Children of Afro-Caribbean, African or Maghrebian descent were more likely to have mental health problems as compared to children of Western descent. This was true for parent-reported conduct disorders and close to significant for internalizing disorders. However, while the trend was also observed for child-reported internalizing disorders, it was not the case for child-reported conduct disorders. The Dominic Interactive has been shown to more accurately detect internalizing disorders as compared to externalizing disorders as children are less likely to report that they experience externalizing problems [22].

Children of mixed couples where one of the parents is from a region of the Caribbean or Africa also had increased risk of mental health problems, though to a lesser extent. Regarding overall difficulties (SDQ ‘total difficulties’), only children of Maghrebian parents displayed elevated scores together with children from single-parent homes. These findings are in contrast with reports suggesting that children of immigrant parents are more likely to have mental health problems [23], though consistent with a report conducted in London using parent-reported child psychopathology suggesting that children of immigrant parents do not differ from children of non-immigrant parents [24].

Another important finding is the evidence of an interaction between punitive behaviors and parental region of birth on child mental health. These results suggest a protective effect of Caribbean or African origin for the negative effects of punitive behaviors on internalizing disorders as reported by the child. Indeed, in the United States it has been observed that, although African Americans (compared to European Americans) exhibit greater authoritarian parenting practices including negative, or harsh control or ‘tough love’, these behaviors are less likely to generate negative outcomes in children possibly because children do not interpret this harshness as a form of rejection, nor as indicative of an ‘out of control’ reaction by the parent [25].

The mental health of children of Maghrebian parents differed from that of Afro-Caribbean or African parents. Although mothers from Maghreb have similar parental behavior to what is observed in women from Western regions, children seem to have more difficulties. Moreover among mothers originating from Maghreb, punitive behavior had a strong negative impact on children, as did low autonomy promoting attitudes which affected internalizing disorders. This finding could indicate that mothers from Maghreb who engage in these behaviors are considerably deviant as they do not conform to their cultural norms. This could be explained by maternal psychopathology, however, in the present study, this result held true when controlling for maternal psychological distress. An alternative explanation might be that although most women from Maghreb are well adapted to French cultural norms, some might remain very isolated and live in communities that are not integrated into French society.

Overall these findings show that what seems to be the most damageable for child mental health is not so much the mother’s parental behavior but the cultural context in which these behaviors are displayed, as ethno-cultural background modulates the child’s expectancies and interpretations of parental behavior.

## Limitations

Several limitations should be considered when interpreting the findings. First, to assess a possible response bias, we compared responding and non-responding parents by school area and characteristics and found limited differences. Children whose parents did not return the questionnaire were more likely to be in a disadvantaged education zone (DSA) (47.8 % vs 59.0 %; *p* < 0.001) but did not differ regarding private/public (*p* = 0.09) or rural/urban school characteristics (*p* = 0.96). Second, parental behaviors were self-reported which might lead to a self-serving underestimation of the prevalence of negative parental attitudes. Parents may not want to disclose their negative attitudes or may fear stigmatization. Furthermore, there might be significant differences in parenting styles perceived by the child vs. reported by the parent. Children often describe their parents as less warm and accepting, and more controlling than the parents describe themselves, and parental reports of their own parenting behavior generally have lower variance than child reports [26]. Third, the region of birth of the current spouse was taken into account, rather than that of the biological father, though in the great majority of the cases, the spouse was the child’s father including once separated though in rare cases the mother could have remarried. Finally, the possibility that the observed differential effects of negative parental attitudes may result from cultural bias in measurement. However, we believe that assessment bias may only be limited as the questionnaire used to assess parental attitudes is based on specific behaviors rather than general attitudes [14] and therefore leaves little room for interpretation. Furthermore, all participants were French residents with a level of fluency in French that allowed them to participate in the study. It is therefore reasonable to believe that language was not an issue in the completion of the questionnaire.

## Conclusion

Parental ethno-cultural background is an important component of both parental behaviors and their effects on children mental health. The implications of these findings are that any intervention geared towards providing parenting support should be culturally relevant to make sense to the parents while at the same time focusing on children particularly at risk. The present study suggests that ethno-cultural context plays an important role in modulating children’s expectations and interpretations of their parents’ behavior. Consequently, interventions might be proposed to inform parents of immigrant origin who exhibit more punitive parenting behavior that this behavior can have negative effects on their child’s mental health as the cultural norms of the country are different. Considering that recent immigrants might oftentimes be isolated, thereby increasing the risk of exhibiting negative parenting behavior, implementing community-based programs where parents could share their experiences raising children and offer one-another advice may help promote a shift towards more positive parenting behavior.

## Ethics approval and consent to participate

The study was approved by the National Committee for Information and Liberty (CNIL authorization number 04-1112). Parents were informed about the study and were given the opportunity to decline participation for themselves and for their child. The authors have full access to all of the study’s original data and take responsibility for their integrity, as for the accuracy of data analysis.

## Consent for publication

Not applicable.

## Availability of data and materials

The data are not made available at this time as they are currently being analyzed for further publications.

## Abbreviations

CMU: Couverture Maladie Universelle: free medical care for socially disadvantaged persons
CNIL: National Committee for Information and Liberty
DI: Dominic Interactive
DSA: Disadvantaged School Areas
PACA: Provence Alpes Cote d’Azur region
PBAQ: Parent Behavior and Attitude Questionnaire
SDQ: Strengths and Difficulties Questionnaire
